# The Ubiquitin-Associated and SH3 Domain-Containing Proteins (UBASH3) Family in Mammalian Development and Immune Response

**DOI:** 10.3390/ijms25031932

**Published:** 2024-02-05

**Authors:** Katarina Vukojević, Violeta Šoljić, Vlatka Martinović, Fila Raguž, Natalija Filipović

**Affiliations:** 1 Department of Anatomy, Histology and Embryology, University of Split School of Medicine, 21000 Split, Croatia; natalija.filipovic@mefst.hr; 2 Department of Anatomy, School of Medicine, University of Mostar, 88000 Mostar, Bosnia and Herzegovina; 3 Department of Histology and Embryology, School of Medicine, University of Mostar, 88000 Mostar, Bosnia and Herzegovina; violeta.soljic@mef.sum.ba; 4 Faculty of Health Studies, University of Mostar, 88000 Mostar, Bosnia and Herzegovina; 5 Center for Translational Research in Biomedicine, University of Split School of Medicine, 21000 Split, Croatia; 6 Department of Surgery, School of Medicine, University of Mostar, 88000 Mostar, Bosnia and Herzegovina; vlatka.martinovic@mef.sum.ba; 7 Department of Internal Medicine, School of Medicine, University of Mostar, 88000 Mostar, Bosnia and Herzegovina; fila.raguz@mef.sum.ba

**Keywords:** UBASH3A, UBASH3B, development, mammals

## Abstract

UBASH3A and UBASH3B are protein families of atypical protein tyrosine phosphatases that function as regulators of various cellular processes during mammalian development. As UBASH3A has only mild phosphatase activity, its regulatory effects are based on the phosphatase-independent mechanisms. On the contrary, UBASH3B has strong phosphatase activity, and the suppression of its receptor signalling is mediated by Syk and Zap-70 kinases. The regulatory functions of UBASH3A and UBASH3B are particularly evident in the lymphoid tissues and kidney development. These tyrosine phosphatases are also known to play key roles in autoimmunity and neoplasms. However, their involvement in mammalian development and its regulatory functions are largely unknown and are discussed in this review.

## 1. Introduction

The UBASH3 family, specifically UBASH3A (previously known as Sts-1) and UBASH3B (previously known as Sts-2), plays crucial roles during mammalian development and immune responses [1]. These proteins contain ubiquitin-associated (UBA) and Src homology 3 (SH3) domains, which are essential for their functions in cellular signalling, particularly in regulating immune cell signalling [2]. Accordingly, they modulate various signalling pathways in immune cells like T-cells, B-cells, and macrophages. In particular, their functions include regulating the activation thresholds of T-cell receptors (TCRs) and B-cell receptors (BCRs), thus influencing the magnitude of immune responses [3]. Additionally, UBASH3 proteins are implicated in maintaining immune tolerance by fine-tuning the signalling cascades in immune cells. The dysregulation of these proteins can lead to over-activation of immune responses, contributing to autoimmune diseases [4]. UBASH3 proteins are associated with regulating inflammatory responses by modulating the activity of key molecules involved in immune signalling pathways. They can influence cytokine production and the activation state of immune cells, thereby affecting the inflammatory microenvironment [5]. However, their roles in development are less studied. UBASH3 proteins have been found to have implications in certain developmental processes [6]. They might influence cell fate decisions or signalling events that are critical for proper embryonic development, although the specific mechanisms are not yet fully understood. Research into the UBASH3 family of proteins continues to uncover their significance in various cellular processes, particularly in the context of immune function and development. Understanding their precise roles in these pathways is crucial for developing potential therapeutic interventions targeting immune-related disorders and diseases.

## 2. UBASH3 Homology and Structures

**Figure 1:**
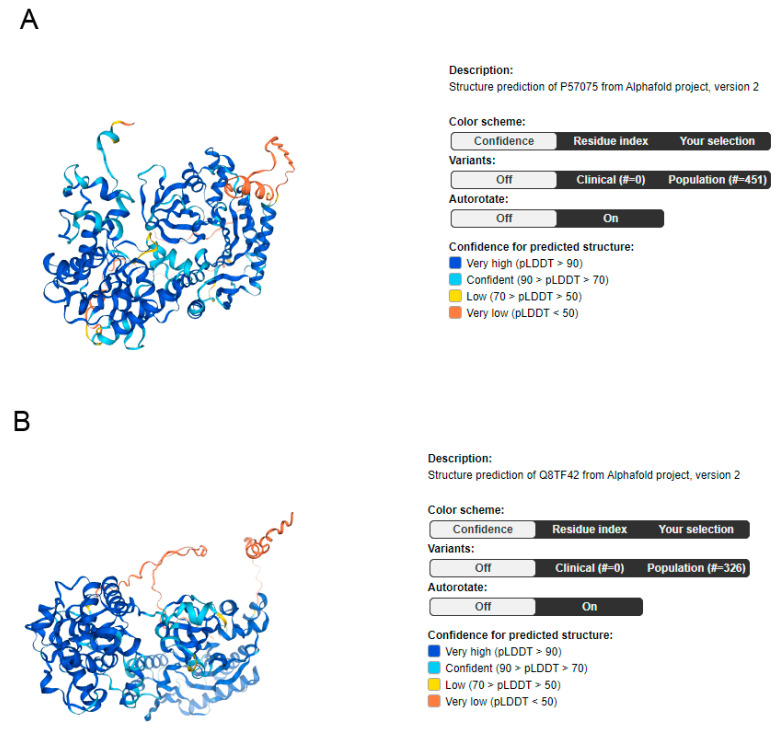
Illustrations of UBASH3A (**A**) and UBASH3b (**B**), detailing their respective domains and confidence in predicting structure. image available from v23.0.proteinatlas.org. Human Protein Atlas https://www.proteinatlas.org/ENSG00000160185-UBASH3A/structure (accessed on 29 January 2024).

UBASH3 stands for ubiquitin-associated (UBA) and Src-homology 3 (SH3) domain. The UBASH3 two-member family is encoded by the UBASH3A and UBASH3B genes and serves as atypical protein tyrosine phosphatases [7]. UBASH3 length is 624 amino acids (aa), and its mass is 70,145 Daltons (Da). The protein attributes for the UBASH3A gene are 661 aa, whereas the molecular mass is 74,123 Da (Figure 1A) [8]. The protein attributes for the UBASH3B gene are 649 aa, whereas the molecular mass is 72,696 Da (Figure 1B) [8].

The UBASH3A gene is mapped to chromosome 21q22.3, whereas the UBASH3B gene is mapped to chromosome 11q24.1 [9]. The UBASH3 protein family (TULA or STS family) has a unique domain architecture, containing an N-terminal ubiquitin-binding domain (UBA), a centrally located SH3 domain, and a C-terminal histidine phosphatase domain, which is also referred to as a phosphoglycerate mutase-like domain [10]. The UBASH3A and B proteins share a 75% amino acid homology [11]. UBASH3A is the first described protein that contains both UBA and SH3 domains and has three isoforms produced by alternative splicing (Table 1) [8].

Although UBASH3A and B share some structural domains, they have different functions and expression patterns [3]. The UBA domain has been shown to bind to monoubiquitin and ubiquitylated proteins, including UBASH3 family proteins when ubiquitylated [7,12]. The SH3 domain of UBASH3 family proteins mediates binding to other proteins, for example, by interacting with CBL (E3 ubiquitin ligase) [10,13]. The histidine phosphatase domain has the ability to hydrolyze low-molecular-weight phosphatase substrates, i.e., proteins that contain phosphotyrosine and are the main substrates under physiological conditions [14]. The domain closest to the C-terminal end of UBASH3A mediates protein dimerization, but the functional role of oligomerization is unclear [14]. UBASH3A is expressed primarily in T-cells and encodes a protein called ubiquitin-associated and SH3 domain containing A [3]. UBASH3A gene expression is restricted to lymphoid tissues, specifically T-cells, and it is localized in the nucleoplasm and the Golgi apparatus in both humans and mice. Therefore, UBASH3A is regarded as a lymphoid protein, unlike its UBASH3B paralog, which is ubiquitous [3]. UBASH3B is expressed in the cytoplasm, nucleoplasm, and nuclear bodies of several tissues, particularly lymphoid tissues, in both humans and mice. UBASH3B and, to some extent, UBASH3A dephosphorylate tyrosine-phosphorylated proteins, which is their key contribution to the molecular basis of their regulatory effect [7]. The UBASH3 gene is not exclusive to mammals and its orthologs are present in other eukaryotes [8]. However, the role of UBASH3A is more intriguing because it provides clear information about its specific functions in mammals. UBASH3A is a negative regulator of T-lymphocyte activation and function [3,4,15]. It is important to control T-cell responses in diseases characterized by an autoimmune and/or chronic inflammatory response.

## 3. UBASH3 Human Disease Models

**Figure 2:**
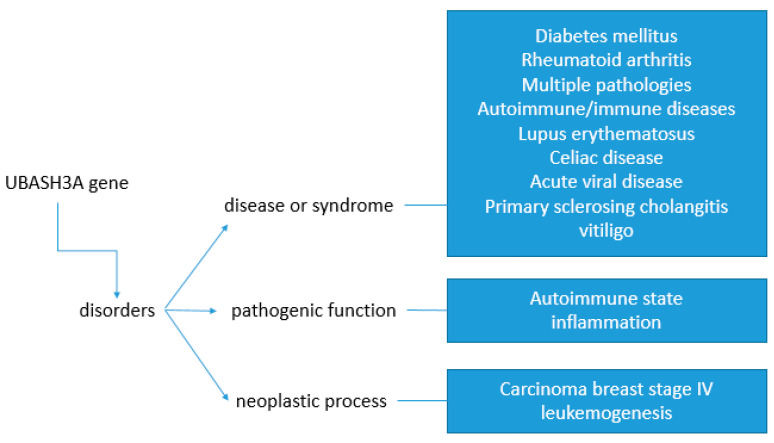
Schematic view of diseases associated with UBASH3A gene UBASH3A gene disruption might lead to disease or syndrome, pathogenic function, or neoplastic process with different consequences on human health.

UBASH3A is a gene that has been linked to type 1 diabetes and other autoimmune diseases [4]. Diseases associated with certain UBASH3A single nucleotide polymorphisms include rheumatoid arthritis [16], vitiligo [17], Graves’ disease [18], celiac disease [16], systemic lupus erythematosus [19,20,21], atopic dermatitis [22], and Addison’s disease [23] (Figure 2).

## 4. UBASH3 Gene Models

The UBASH3A gene downregulates inflammation in the trinitrobenzene sulfonic acid-induced colitis model, where the knockout of either Ubash3a or Ubash3b increases both inflammation and T-cell responses, but the Ubash3a Ubash3b double-knockout mice display a more severe phenotype than either of the single-knockout mice [3]. A single-knockout mouse is phenotypically indistinguishable from a wild-type mouse [24]. UBASH3A deficiency strongly affects the T-lymphocyte function and immune response under physiological and pathological conditions, but the molecular basis of UBASH3A’s effect on cell activation and biological response during development is not yet clear. UBASH3B negatively regulates T-cell receptor (TCR) signalling in activated T-lymphocytes [25]. Additionally, it was shown that in vitro and in vivo, UBASH3B has an important protein tyrosine phosphatase activity that suppresses T-cell receptor signalling via the dephosphorylation of ZAP-70 [26] and Syk [27]. UBASH3B has been found to be a novel prognostic biomarker correlated with immune infiltrates in prostate cancer [25]. However, UBASH3B is not known to be associated with autoimmune or any immune-mediated disorders in genome-wide association studies [4].

## 5. The UBASH3 Signalling Pathway in Mammalian Development

Although there are a significant number of different signalling pathways during mammalian development, only a small number of signalling pathways are necessary for cell–cell interactions during embryonic development. However, the preservation of signals and mechanisms throughout evolution enables the repeated use of the same signalling pathways at different times and places during mammalian development. Understanding these signalling pathways provides us with information about cell functioning and the interactions needed for proper organ morphogenesis. These findings can also reveal the underlying basis of congenital anomalies since the disturbance of cell signalling during embryonic development is the main cause of malformations. Additionally, these findings can help in the identification of underlying causes of neoplasms and serve as potential therapeutic targets. Signalling molecules that play a major role in the patterning of the developing embryos are capable of significantly affecting proper mammalian development. These signalling pathways include the Wnt, transforming growth factor-beta (TGFβ), Hedgehog, Notch, receptor tyrosine kinase (RTK), JAK/STAT, and nuclear hormone pathways [28]. The Wnt signalling pathway is a group of signal transduction pathways that regulate crucial aspects of cell fate determination, cell migration, cell polarity, neural patterning, and organogenesis during embryonic development [29]. Transforming Growth Factor-beta is a multifunctional protein that regulates the growth and differentiation of various cell types and is involved in various cellular processes [30]. Notch signalling is important for cell-to-cell communication pathways that control stem cell fates, such as self-renewal, proliferation, and differentiation during early embryonic development and postnatal life [31]. The JAK/STAT signalling pathway plays an important role in transmitting extracellular signals and has been shown to play a role in the regulation of T-cell function and activation [32]. Receptor tyrosine kinases are a family of cell surface receptors that play crucial roles in cell growth, differentiation, and survival, and their dysregulation has been implicated in cancer [33]. Ubiquitin-mediated mechanisms are directly or indirectly implicated in many of these pathways and have been linked to various processes, including ubiquitination events [34]. During evolution, these pathways are advanced within the cell cycle in order to produce cellular and morphological developmental diversity, but during aging disturbances in the cell cycle are linked to various diseases in humans. The cell cycle includes many key factors and enzymes that require precise spatiotemporal regulation. Although ubiquitination is the main selective system for protein degradation, the cell requires ubiquitination for various biological processes including cell division [35]. Special emphasis is placed on the UBDs (ubiquitin-binding proteins) that mediate cellular fates and downstream signalling by interacting with ubiquitylated substrates. UBASH3A as one of such substrates stimulates activated target receptors, such as EGFR and PDGFRB, on the cell surface [3]. UBASH3A has also been found to regulate the synthesis and dynamics of TCR-CD3 complexes [3]. In addition, UBASH3A interferes with the CBL-mediated downregulation and degradation of receptor-type tyrosine kinases, promoting the accumulation of activated target receptors [13]. Reppschlager et al. reported that UBASH3B dephosphorylates various pY-containing proteins in vitro, including several protein tyrosine kinases such as Src, Zap-70, EGFR, Cbl, and Syk [36]. Additionally, UBASH3A is involved in the regulation of Syk cellular signalling [7]. These UBASH3 activities reduce their tyrosine phosphorylation and their phosphorylation in the cells. The UBASH3 substrate spectrum in the developmental context may also be affected by the SH3 domain, which mediates binding to proline-rich motif-containing SH3-binding proteins [7]. Protein–protein interactions are involved in many cellular processes, some of which are directed by SH3 domains that bind proline-rich motifs to other proteins. However, the binding specificity of SH3 domains is poorly understood. Additionally, UBASH3A hinders dynamin-dependent endocytic pathways by sequestering dynamin through its SH3 domain [37]. Additionally, UBASH3A inhibits Cbl via direct interaction and thus inhibits the suppression of EGFR signalling, which is important during mammalian development [38]. The UBASH3 signalling pathway, particularly the UBASH3B protein, plays a crucial role in mitosis by localizing Aurora B to the microtubules, ensuring proper chromosome segregation [39]. This pathway is also involved in the regulation of fundamental processes in mammalian stem and progenitor cells, including development, survival, and differentiation [40]. Accordingly, UBASH3 loss- and gain-of=-function have important effects on mitotic progression.

The UBASH3A gene, which encodes a potential nuclear protein, is expressed in various tissues, with the highest levels found in the spleen, peripheral blood leukocytes, and bone marrow [41], but its role during mammalian development is still not clear. Stamenova et al. identified a subset of SH3 domains that bind to ubiquitin, a key protein in the ubiquitin-proteasome system. A subset of SH3 domains constitutes a distinct type of ubiquitin-binding domain, and therefore, ubiquitin-binding can negatively regulate the interaction of SH3 domains with canonical proline-rich ligands [42]. This suggests a potential role for UBASH3A in the regulation of protein interactions and the ubiquitin-proteasome system, which is crucial for cellular homeostasis and development. There is only one paper by Lozic et al. from our group, who describe UBASH3A expression patterns in different structures of the developing human kidney [6]. We found that the UBASH3A protein was observed as punctate, with mostly nuclear staining in developmental kidney tubules and glomeruli. Metanephric mesenchyme-derived developmental structures were markedly more positive than the mature tubules. This only finding of UBASH3A during human kidney development from the 13th week to the 1st year and a half postnatal period indicates UBASH3A involvement as a renal development control gene. The analyzed kidney structures exhibited insignificant fluctuations in UBASH3A cell immunoreactivity during development and postnatally, whereas the analysis of area percentage showed greater expression of UBASH3A protein in the prenatal phase where maximum growth of the fetus was achieved. In vitro UBASH3A gene knockdown studies on a renal cell carcinoma cell line showed inhibited cell migration and inhibited viability, implying the potential for metastasis induction in clear cell renal cell carcinoma [43]. Therefore, this study could provide a missing link that helps link together UBASH3A and congenital anomalies of the kidney and urinary tract. Considering the fact that the regulatory effect of UBASH3A is still not very well understood, cell regulation of a spleen tyrosine kinase (Syk), which plays a role in receptor signal transduction, is the best characterized effect of UBASH3 family proteins on signalling [7]. UBASH3 proteins were shown to interact with Syk and regulate its activity, with UBASH3A being identified as a negative regulator of mast cell degranulation, a process involving the release of granules from mast cells. Additionally, the non-receptor tyrosine kinase Syk has been identified as a target of Cbl-mediated ubiquitylation upon B-cell receptor stimulation [44]. This suggests that the UBASH3 protein family, including UBASH3A and UBASH3B, plays a role in the regulation of Syk activity and may be involved in the modulation of various cellular processes, including mast cell degranulation and B-cell receptor signalling. Through its upregulation of Syk, UBASH3A may play a role during kidney development through the GDNF/Ret and PI3K pathways, which are crucial in the context of normal branching morphogenesis. Additionally, UBASH3A is a Cbl-interacting protein and, in that regard, UBASH3A was found to inhibit Cbl in order to suppress EGFR signalling [13]. Since it has been known for a very long time that the CRKL SH2 domain binds to Cbl [45], it might be possible that CRKL and UBASH3A share a common signalling pathway that involves EGFR signalling. This explains the similarities in their expression patterns. CRKL expression resembles that of UBASH3A with the highest expression in embryogenesis, which would link it together with the genes controlling spatial and temporal distribution [46]. However, the mechanism underlying this involvement has not been investigated. Nevertheless, further research may be needed to fully understand the mechanisms by which the UBASH protein family regulates Syk activity and its potential implications in health and disease. Jinwoo Ahn et al. found that UBASH3A was commonly mutated in patients with clear cell renal cell carcinoma and was thought to be a metastasis-associated candidate gene [43]. This finding supports the UBASH3A involvement in both kidney development and neoplasm. However, further studies are needed to elucidate the mechanism of its involvement in mammalian development, kidney physiology, and pathology.

**Figure 3:**
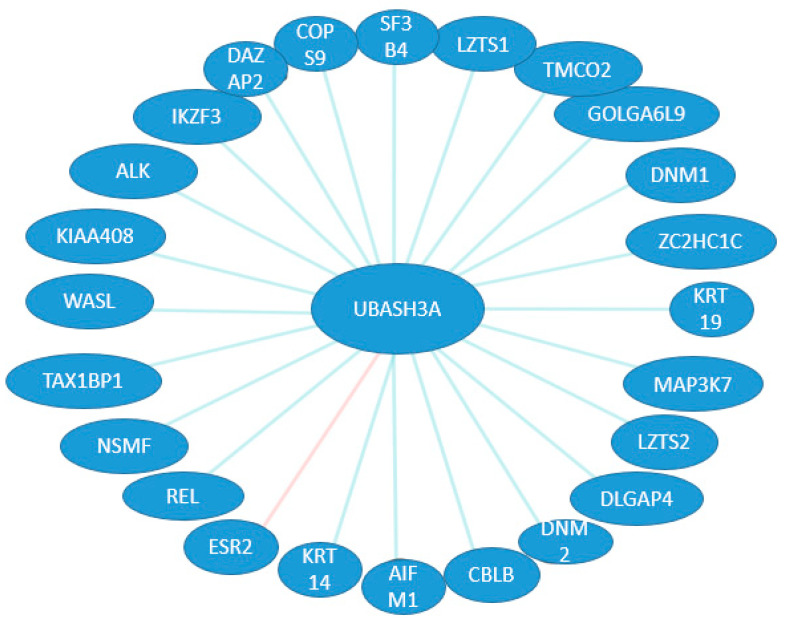
Possible binding and expression patterns of UBASH3A and its associations with other proteins. Binding (blue line) and expression (pink line) interactions between UBASH3A and other genes.

**Figure 4:**
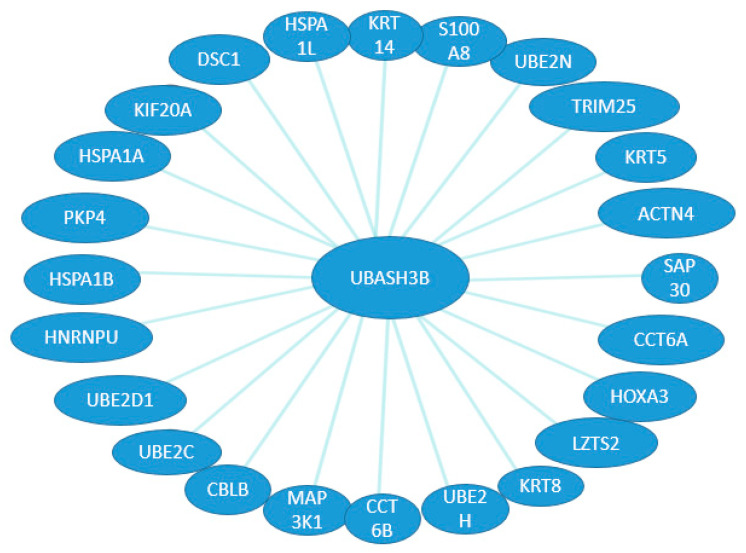
Possible binding and expression patterns of UBASH3B and its associations with other proteins. Binding (blue line) interactions between UBASH3B and other genes.

On the basis of available databases about the possible binding and expression patterns of UBASH3 family members and their associations with other proteins, we can speculate on their interactions in different organ systems during development. So far, there are 23 binding sites and one expression interaction (Figure 3) for UBASH3A, whereas for UBASH3B, there are 24 binding sites known (Figure 4). However, searching the literature has led to finding a missing gap in the functional characterization of these interactions during development.

However, the intricate interplay of UBASH3 proteins within signalling networks highlights their versatility in modulating cellular pathways that are critical for proper development. Their regulatory influence on pivotal signalling cascades, including those involving receptor tyrosine kinases and immune receptors, emphasizes their indispensable contribution to the orchestration of developmental processes.

## 6. The Role of UBASH3 in Apoptosis and Autophagy

**Figure 5:**
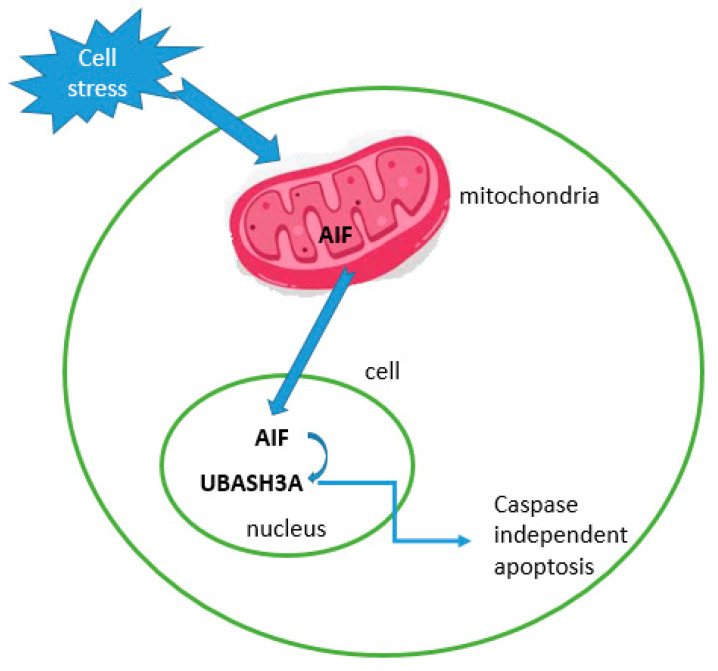
Cartoon illustration of how UBASH3A interacts with AIF and its downstream effects.

The role of UBASH3 in apoptosis is part of a larger network of proteins and pathways that regulate this process. Using RNA interference, Collingwood et al. demonstrated that apoptosis-inducing factor (AIF) is essential for the apoptotic effect of UBASH3A. T-cell ubiquitin ligands affect cell death through a functional interaction with AIF, a key factor in caspase-independent apoptosis [47,48]. UBASH3A binds to AIF, which is released from the mitochondria and translocates to the nucleus when the cell is exposed to environmental stress, and this translocation leads to caspase-independent apoptosis. In the nucleus, UBASH3A binds to AIF. UBASH3A is one of the few functional interactors of AIF. It enhances AIF-dependent apoptotic events and is not an independent inducer of apoptosis. The said effect depends on its UBA and SH3 domains [47]. Additionally, UBASH3A can enable growth factor withdrawal-induced apoptosis in T-cells by interacting with AIF. Alternative splicing of this gene results in multiple transcript variants [49]. Therefore, the involvement of UBASH3A in caspase-independent apoptosis through its interaction with AIF might be one of the key regulators of this evolutionary oldest apoptotic pathway (Figure 5). In many examples of mammalian cell death, the cell developmental fate between death and life is upstream or independent of caspase activation [50].

Ubash3b plays an inhibitory role in BCR-ABL signalling by binding and dephosphorylating BCR-ABL and its interactors [51]. Ubash3b displays a strong negative regulatory role by dephosphorylation of p210 and p210 signalling molecules in addition to its effects on proteins that interact with p210 [51]. Additionally, the downregulation of UBASH3B can further potentiate the Spindle Assembly Checkpoint response inducing mitotic arrest and cell death in cancer cells expressing high levels of UBASH3B and Spindle Assembly Checkpoint proteins [52]. These studies indicate a role for Ubash3b in tumor suppression, which might consider Ubash3b as an attractive therapeutic target for malignancies driven by active tyrosine kinases.

**Figure 6:**
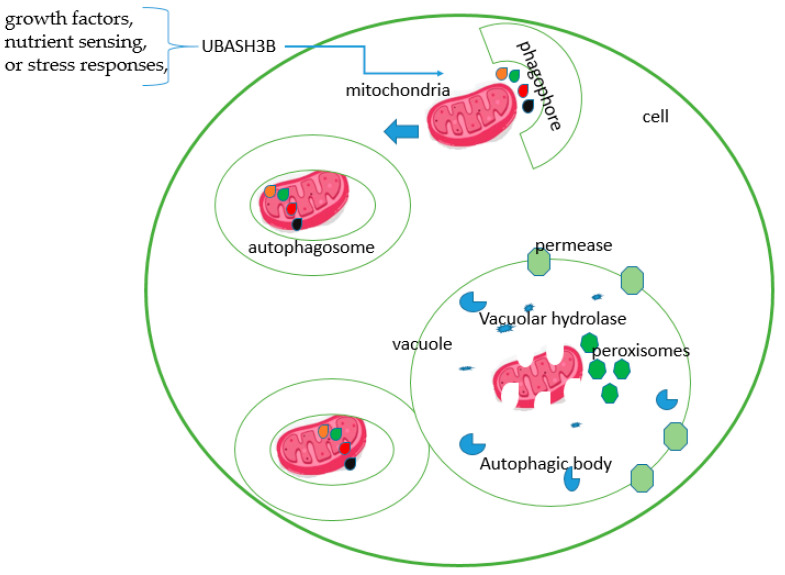
Cartoon illustration of how UBASH3B is involved in the autophagic process.

The UBASH3 protein family has garnered attention because of its multifaceted involvement in cellular processes, including its emerging association with autophagy [53]. Autophagy, a fundamental cellular mechanism, orchestrates the degradation and recycling of cellular components, contributing significantly to cellular homeostasis, development, and disease pathogenesis [54]. Recent studies suggest a potential interplay between UBASH3 proteins and autophagic pathways [55]. UBASH3 proteins are known to modulate intracellular signalling by regulating the ubiquitination and degradation of specific proteins involved in various cellular functions. In this context, their interaction with key autophagy-related proteins or signalling components might influence the autophagic process (Figure 6).

In particular, UBASH3 proteins could potentially affect autophagy by modulating the turnover of critical regulators or effectors within the autophagic machinery. Their role in regulating signalling pathways that intersect with autophagy, such as those involved in growth factors, nutrient sensing, or stress responses, further implies their potential influence on autophagic processes [7]. However, the direct mechanisms by which UBASH3 proteins contribute to autophagy and the precise interconnections between UBASH3 family members and autophagic components remain areas of ongoing research. Continued investigation into the crosstalk between UBASH3 proteins and autophagy holds promise for uncovering novel regulatory mechanisms and potential therapeutic targets, enhancing our understanding of cellular homeostasis, and paving the way for innovative approaches to treat diseases associated with autophagic dysregulation.

## 7. The UBASH3 Proteins in Inflammation

The UBASH3 proteins, particularly UBASH3A and UBASH3B, play crucial roles in inflammation and immune responses. T-cells are key players in the process of eliminating foreign pathogens within the mammalian immune system. In this process, they directly eliminate cells that contain pathogens or they secrete different cytokines that activate other cell types [27]. The activity of T-cells is largely controlled by the T-cell receptors (TCRs). TCRs are associated with transmembrane proteins containing immune-receptor tyrosine-based activation motifs (ITAMs). The TCR distinguishes the presence of pathogens and activates numerous intracellular signalling pathways downstream of the TCR [56]. ITAMs are phosphorylated upon receptor engagement, which leads to the recruitment and activation of SYK or the related ζ-chain-associated protein kinase of 70 kDa (ZAP70).

**Figure 7:**
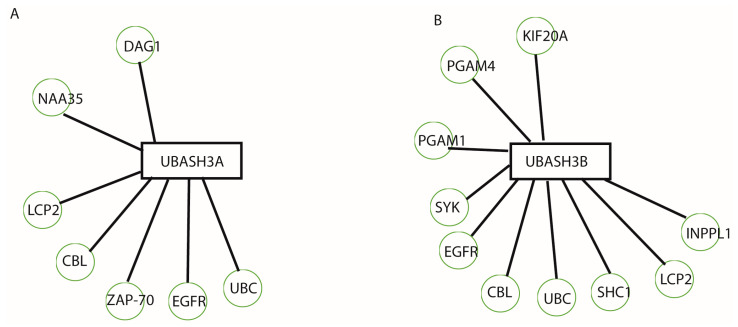
UBASHA (**A**) and UBASHB (**B**) experimentally determined protein interactions [57].

Both UBASH3A and UBASH3B function as negative regulators of TCR signalling in order to prevent extreme and damaging T-cell responses. This regulation is achieved by a phosphoglycerate mutase-like domain. Additionally, UBASH3A hampers the CBL-mediated downregulation of receptor-type tyrosine kinases. UBASH3A regulates T-cell tyrosine kinase Zap-70 (Figure 7A) and therefore tyrosine phosphorylation on targets within T-cells, implying phosphatase activity and involvement in setting up the threshold for T-cell activation through TCR [27]. Hu et al. suggested that the ubiquitination of Zap70 may negatively regulate Zap70 activity by recruiting the protein tyrosine phosphatase UBASH3A and its homolog, UBASH3B [5]. UBASH3B also exhibits tyrosine phosphatase activity toward a few proteins, such as EGFR, KIF20A SYK, and ZAP70 (Figure 7B).

The UBASH3 protein’s involvement in inflammation has been well studied in various autoimmune diseases. The transcription of UBASH3A in rheumatoid arthritis is suppressed via the epigenetic regulation of super-enhancers in CD4+ T-cells. Low UBASH3A levels result in excessive TCR signal activation with subsequent enhancement of IL-6 production [58]. Additionally, Ge et al. revealed that UBASH3A reduces the T-cell receptor-induced NF-κB signalling via a ubiquitin-dependent mechanism upon TCR stimulation, which plays a key role in innate and adaptive immune responses [4]. This signalling suppresses the IκB kinase complex activation, resulting in IL2 gene expression reduction [4]. The effect of UBASH3A on IL-2 production is mediated by NF-κB and UBASH3A acts upstream of the nuclear translocation of NF-κB [4]. The increased activity of the IKK complex in TCR-stimulated UBASH3A cells, without comparable increases in the activation of the upstream signalling molecules PKCδ and TAK1, suggests that UBASH3A specifically suppresses the activation of the IKK complex [4]. These findings suggest that the NF-κB signalling pathway is regulated by UBASH3A. Additionally, when human erythroleukemia cell lines were investigated for different drug compounds, it was revealed that Ubash3b expression was induced by TPA (4β-12-O-tetradecanoylphobol-13-acetate), which led to PKCδ protein dephosphorylation and degradation [59]. On the contrary, this degradation was blocked by the RNAi-mediated depletion of Ubash3b. Therefore, specific inhibitors of Ubash3b can overcome resistance to TPA, leading to long-lasting suppression of leukemic growth [59]. Additionally, UBASH3B has been identified as a potential prognostic biomarker in prostate cancer and is associated with tumor-infiltrating immune cells [25]. The nuanced functions of UBASH3 proteins in fine-tuning immune responses and maintaining immune homeostasis underscore their importance beyond developmental stages, implicating their involvement in disease pathogenesis and their therapeutic potential. These findings highlight the significance of UBASH3 proteins in immune-related diseases and their potential as therapeutic targets.

## 8. The UBASH3 Proteins in Autoimmunity

While UBASH3B’s role in autoimmunity appears to be limited, UBASH3A has been associated with several autoimmune diseases, particularly in the regulation of T-cell function in the context of type 1 diabetes, systemic lupus erythematosus, celiac disease, rheumatoid arthritis, and vitiligo [4,19,20,25]. Liu et al. showed that UBASH3A gene polymorphisms are associated with systemic lupus erythematosus in a Chinese population [20]. UBASH3A has been shown to regulate T-cell activation and T-cell receptor CD3 complex turnover, contributing to autoimmunity [3]. Although the exact molecular mechanism associated with this gene mutation in type 1 diabetes is largely unknown, Ge et al. suggested that UBASH3A, as a negative regulator of T-cell function, inhibits its induction of NF-κB signalling, thereby affecting the expression of critical genes involved in autoimmunity, such as IL2 [3]. UBASH3A regulates the NF-κB signalling pathway via a ubiquitin-dependent mechanism. Additionally, two type 1 diabetes-associated variants (risk alleles at rs11203203 and rs80054410) in UBASH3A increase UBASH3A expression in primary CD4+ T-cells in humans after T-cell receptor stimulation, which inhibits NF-κB signalling through its effects on the IκB kinase complex, resulting in reduced IL2 gene expression [4]. Therefore, UBASH3A might be involved in mediating the risk of type 1 diabetes by inhibiting T-cell receptor-induced NF-κB signalling. Additionally, Chen et al. also demonstrated that UBASH3A deficiency accelerates type 1 diabetes development and enhances salivary gland inflammation, further highlighting its broad impact on autoimmunity [60]. Although UBASH3A has been identified as a potential therapeutic target, there are no clinical trials investigating its use in treating autoimmune diseases. However, it is important to further investigate UBASH3A as a potential therapeutic target due to UBASH3A’s role in autoimmunity through the regulation of T-cell function and the modulation of signalling pathways that are critical in autoimmune diseases.

## 9. Conclusions

The ubiquitin-associated and SH3 domain-containing protein (UBASH3) family is a crucial player during mammalian development, orchestrating diverse cellular processes through its regulatory functions. Across various developmental stages, UBASH3 proteins exhibit multifaceted roles in signal transduction, immune regulation, and cellular homeostasis. Their interaction with key signalling molecules and their involvement in pathways associated with growth, differentiation, and immune responses underscore their significance in shaping mammalian development.

The available literature provides us with information to conclude that UBASH3A and UBASHB are key regulators of cellular functions. Their SH2 domains detect phosphorylated tyrosine residues; therefore, several physiological and molecular pathways in mammalian cells are regulated by their signalling domains. Any dysregulation of their SH3 domains might be involved in the onset of different disorders and diseases. Advances in understanding the role of Tula-family proteins in cell signalling and activation include the identification of novel regulatory functions of UBASH3 proteins in the context of platelets, where UBASH3B negatively regulates platelet signalling mediated by ITAM- and hemITAM-containing membrane receptors. Second, UBASH3B activates the JAK1-STAT1 pathway, facilitating autophagy in an interferon-α signalling context. Challenges in studying UBASH family proteins include resolving the molecular basis of UBASH3 proteins’ effects, including targets of regulatory dephosphorylation in their already known and novel substrates, phosphatase-independent mechanisms, and the individual specificity of the two family members UBASH3A and UBASH3B.

In this review paper, we summarized the current knowledge about UBASH3A and UBASHB in mammalian development, as well as their roles in apoptosis, inflammation, and autoimmunity. Crosstalk between UBASH3A, UBASHB, and developmental signalling pathways produce the diverse cellular behaviors that are needed to build lymphoid tissues and organs, such as the kidneys. The mechanisms by which UBASH3A and UBASHB regulate these molecular pathways remain unclear. It is well known that UBASH3A is primarily expressed in T-cells and is associated with autoimmune diseases, whereas UBASH3B is ubiquitously expressed and involved in regulating T-cell receptor signalling and prostate cancer. Despite the advances in understanding the roles of UBASH3 family proteins in cell signalling and activation, there is still much to learn about their precise mechanisms in the early embryonic events that might represent a basic step toward a better understanding of the effect of pathological mutations in UBASH3A and UBASHB genes and, consequently, to the design of therapeutical targets aimed at regulating their malfunctions, particularly in the context of inflammation and autoimmune diseases. Therefore, further elucidation of the specific molecular mechanisms and intricate interactions mediated by UBASH3 proteins will undoubtedly deepen our understanding of their contributions to mammalian development and potentially unveil novel avenues for therapeutic interventions targeting developmental disorders and immune-related diseases. Continued research on the UBASH3 family holds promise for uncovering new insights into the intricate landscape of mammalian development and its implications for health and disease.

## Figures and Tables

**Table 1:** UBASH3A and UBASH3B splice variants, SwissProt, TrEMBL, protein class, length, mass, predicted signal peptide, and predicted transmembrane regions.

Splice Variant	SwissProt	TrEMBL	Protein Class	Length and Mass	Signal Peptide (Predicted)	Transmembrane Regions (Predicted)
UBASH3B-201	Q8TF42		Enzymes Predicted intracellular proteins Mapped to neXtProt Protein evidence [9] Protein evidence [10]	649 aa 72.7 kDa	No	0
UBASH3A-201	P57075 [Direct mapping] Ubiquitin-associated and SH3 domain-containing protein A		Predicted intracellular proteins Mapped to neXtProt Protein evidence [9] Protein evidence [10]	623 aa 69.8 kDa	No	0
UBASH3A-202	P57075 [Direct mapping] Ubiquitin-associated and SH3 domain-containing protein A		Predicted intracellular proteins Mapped to neXtProt Protein evidence [9] Protein evidence [10]	661 aa 74.1 kDa	No	0
UBASH3A-203	P57075 [Direct mapping] Ubiquitin-associated and SH3 domain-containing protein A		Predicted intracellular proteins Mapped to neXtProt Protein evidence [9] Protein evidence [10]	526 aa 59.2 kDa	No	0
UBASH3A-208		A0A0U1RQL4 [Direct mapping] Ubiquitin-associated and SH3 domain-containing protein A	Predicted intracellular proteins Protein evidence [10]	169 aa 18.7 kDa	No	0

data available from v23.0.proteinatlas.org. Human Protein Atlas https://www.proteinatlas.org/ENSG00000160185-UBASH3A/structure and https://www.proteinatlas.org/ENSG00000154127-UBASH3B/structure (accessed on 29 January 2024).

## Data Availability

Not applicable.
